# Prediction of post-operative atrial fibrillation in patients after cardiac surgery using heart rate variability

**DOI:** 10.1186/s12872-023-03309-5

**Published:** 2023-06-07

**Authors:** Jana Veselá, Pavel Osmančík, Dalibor Heřman, Sabri Hassouna, Radka Raková, Tomáš Veselý, Petr Budera

**Affiliations:** 1 Department of Cardiology, Third Faculty of Medicine, University Hospital Kralovske Vinohrady, Charles University, Ruská 87, Prague, 100 00 Czech Republic; 2 Department of Information and Communication Technologies in Medicine, Faculty of biomedical engineering, Czech Technical University in Prague, Prague, Czech Republic; 3 Cardiac Surgery Clinic, Third Faculty of Medicine, University Hospital Kralovske Vinohrady, Charles University, Prague, Czech Republic

**Keywords:** Post-operative atrial fibrillation, Cardiac surgery, Heart rate variability, Non-linear analysis

## Abstract

**Purpose:**

Post-operative atrial fibrillation (PoAF) occurs in ~ 30% of patients after cardiac surgery. The etiology of PoAF is complex, but a disbalance in autonomic systems plays an important role. The goal of this study was to assess whether pre-operative heart rate variability analysis can predict the risk of PoAF.

**Methods:**

Patients without a history of AF with an indication for cardiac surgery were included. Two-hour ECG recordings one day before surgery was used for the HRV analysis. Univariate and multivariate logistic regression, including all HRV parameters, their combination, and clinical variables, were calculated to find the best predictive model for post-operative AF.

**Results:**

One hundred and thirty-seven patients (33 women) were enrolled in the study. PoAF occurred in 48 patients (35%, AF group); the remaining 89 patients were in the NoAF group. AF patients were significantly older (69.1 ± 8.6 vs. 63.4 ± 10.5 yrs., p = 0.002), and had higher CHA_2_DS_2_-VASc score (3 ± 1.4 vs. 2.5 ± 1.3, p = 0.01). In the multivariate regression model, parameters independently associated with higher risk of AF were pNN50, TINN, absolute power VLF, LF and HF, total power, SD2, and the Porta index. A combination of clinical variables with HRV parameters in the ROC analysis achieved an AUC of 0.86, a sensitivity of 0.95, and a specificity of 0.57 and was more effective in PoAF prediction than a combination of clinical variables alone.

**Conclusion:**

A combination of several HRV parameters is helpful in predicting the risk of PoAF. Attenuation of heart rate variability increases the risk for PoAF.

## Introduction

Post-operative atrial fibrillation (PoAF) is a common complication during the early post-operative period after cardiac surgery. PoAF prolongs hospital stays and affects the subsequent post-operative recovery and prognosis. PoAF is associated with an increased risk of hospital readmission, stroke, and early and late mortality. Depending on the type of procedure, PoAF occurs in 30–60% of patients without a history of atrial fibrillation (AF). The incidence of PoAF is higher in patients undergoing valve surgery than in those undergoing coronary artery bypass grafting (CABG) [1–3].

The exact mechanism causing PoAF has not been fully elucidated. It is generally assumed that it is an interplay of individual factors, which can be divided into acute (directly related to the surgery, e.g., activation of inflammatory complement) and chronic, which are associated with heart aging and remodeling [2]. Several conditions are recognized as risk factors for PoAF in patients without a previous history of AF. The most important include decreased left ventricular ejection fraction, age, left atrial enlargement, chronic obstructive pulmonary disease, peripheral vascular disease, obesity, hypertension, and diabetes mellitus [3, 4].

Importantly, sympathetic and parasympathetic activity (or a disbalance between them) also plays an essential role in AF induction, particularly in patients after surgery, when increased sympathetic activity is present. Post-operative heart rate variability (HRV) studies have shown an increase in time and frequency parameters compared to pre-operative values. However, whether these changes are associated with increased sympathetic activity or loss of vagal tone is not entirely clear [2].

Heart rate variability (HRV) analysis is a commonly used method for evaluating autonomic nervous system activity. HRV measurement is simple, non-invasive, and reproducible when performed under standardized conditions. Several studies have been published on PoAF following cardiac surgery, but few focused on the usefulness of HRV in PoAF prediction. This study aimed to determine if a comprehensive analysis of pre-operative heart rate variability could be used to assess the risk of PoAF. Identification of parameters associated with new-onset PoAF would be of great clinical importance. It would ensure adequate precautions during the perioperative period to optimize clinical outcomes.

## Methods

### Patients and surgery

Patients without an AF history and indicated for cardiac surgery were included. The inclusion criteria were an indication for cardiac surgery due to coronary artery disease or valve disease, absence of an AF history, and sinus rhythm on admission. Exclusion criteria were urgent cardiac surgery, non-sinus rhythm on admission, the presence of an implanted pacemaker or defibrillator, a history of atrial fibrillation, and unwillingness to participate. All surgeries were completed at the Cardiac Surgery Clinic of the University Hospital Kralovske Vinohrady.

All surgical procedures were performed under general anesthesia from a median sternotomy or, in selected cases, an upper partial sternotomy. Most procedures used an extracorporeal circulation device and cardioplegic cardiac arrest; rarely were cardiopulmonary artery bypass graftings without extracorporeal circulation. The use of extracorporeal circulation in CABG surgery was left to the discretion of the lead surgeon. After surgery, all patients were hospitalized in the intensive care unit (ICU) of the Cardiac Surgery Clinic. According to normal clinical practice, extubation was performed after stabilizing respiratory, circulatory, and other parameters and a gradual return of consciousness, usually within a few hours after the procedure. ECGs were monitored throughout the stay in the ICU. After extubation and in stable hemodynamic condition, patients were transferred to the standard post-operative department with facilities for continuous ECG monitoring.

Post-operative AF was defined as a supraventricular arrhythmia with irregular atrial activity lasting longer than 30 s [5] recorded during the first five days after surgery. Based on the post-operative occurrence of AF during the five days after surgery, patients were divided into the AF group and the NoAF group. All collected variables, including the results of the HRV analysis, were compared between the two groups.

### ECG recording for HRV analysis

A detailed patient history was collected from all patients, with the results of blood tests (biochemistry, blood count) and ECGs performed one day before surgery. For the HRV analysis, 2-hour ECGs were recorded in all patients the day before surgery. The ECG recordings took place in a supine position in the afternoon prior to the day of surgery while the patient was entirely at rest.

ECGs were recorded using the VLV system created by the Faculty of Biomedical Engineering, Czech Technical University. It is a multifunctional biotelemetry system to help monitor psychophysiological states. It enables simultaneous and continuous mobile sensing of multiple physiological parameters. It can record ECGs, respiratory curves, skin resistance, myopotentials, and physical activity. The system consists of a scanning unit, a translation unit (a Wi-Fi access point), and one control and collection unit (notebook). It transmits a full stream of digitized signals, not just the calculated parameters, which allows for the preservation of all information for further detailed analysis and research. For our purposes, the system was modified to scan two ECG channels.

Leads were placed in the third intercostal space to the left of the sternum; leads V2, V5, and V6 were placed as usual. Thus, the system scanned two bipolar ECG leads for 2 h. The evaluation of the measured data took place offline. The information was stored in a text file with the “.csv” extension (Comma-separated values). A specially created program transformed these files into a form that could be used by the Kubios HRV (Kuopio, Finland) software. HRV analysis was performed following the recommendations of the European Society of Cardiology and the North American Society of Pacing Electrophysiology [6].

In the Kubios HRV program, all recordings were visually inspected and checked before analysis. This was done to ensure that all normal QRS complexes were detected correctly and atrial or ventricular premature activities, as well as artifacts, were excluded from the analysis. Subsequently, the HRV analysis was performed in the time and frequency domains, and the non-linear analysis was performed. In the frequency-domain methods, a power spectrum density (PSD) estimate was calculated using parametric autoregressive (AR) models. The generalized frequency bands were very low (VLF, 0–0.04 Hz), low (LF, 0.04–0.15 Hz), and high-frequency bands (HF 0.15–0.4 Hz) [7]. Measurements in the time and frequency domain were often insufficient to describe the system’s dynamics, so we also used non-linear methods and models. Non-linear measurements describe the unpredictability of a time series that results from the complexity of the mechanisms that regulate HRV [8]. For the non-linear analysis, we used parameters obtained from the Poincare plot (Table 4). Each RR interval is displayed as a function of the previous RR interval. Several indices can be obtained through fundamental quantitative analysis of Poincare maps, e.g., standard deviation SD1 and standard deviation SD2. SD1 is associated with the rapid variability of successive RR intervals; on the other hand, SD2 describes the long-term variability of RR intervals. The SD1/SD2 ratio can also be determined from these two parameters describing their mutual relationship. This quantitative analysis method is based on the principle of different temporal effects due to changes in vagal and sympathetic modulation of the heart rate. From a physiological point of view, the SD1 parameter describes sharp changes in RR intervals because the vagal effect on the SA node is faster than the sympathetic influence. Both sympathetic and parasympathetic tone affects the SD2 parameter [9, 10]. In addition to the basic parameters, SD1, SD2, and SD1/SD2, the Guzik index [11], the Porta index [12], and the Slope index [11] were calculated; special software was created to do these computations. Poincare maps describe heart rate asymmetry. The Guzik, Porta, and Slope indices describe the properties of RR intervals above and below the identity line. They can therefore determine whether accelerating or decelerating runs predominate in the ECG recording. The Porta index is defined as the percentage of the number of points below the identity line with respect to the total number of points in the Poincare plot. A Porta index > 50 indicates that the bradycardic runs are shorter than the tachycardic runs. Guzik’s index is defined as the percentage of the distance contributed by the points above the identity line with respect to the total distance. The Slope index also considers the phase angle of the points in the Poincare plot [11]. Another approach is the recurrence plot analysis for analyzing the complexity of the time series when the recurrence rate (REC) is obtained. Kubios HRV can also calculate the Approximate entropy (ApEn) and Sample entropy (SampEn). Entropy measures the complexity or irregularity of the signals [7].

### Statistical analysis

Statistical analysis was performed using software R (version 4.1.2 (2021-11-01), The R Foundation for Statistical Computing). Normality was tested using the Shapiro-Wilk test. The groups were compared using the Wilcoxon test when the distribution non-normal. If the quantity met the normality condition, the F-test was used to determine the homogeneity of variances for both groups. If this condition was met, an unpaired two-sample t-test was used to compare groups; if the groups had different variances, the Welch t-test was used. Qualitative variables were tested using Pearson’s chi-squared test. A p < 0.05 was considered statistically significant. Univariate logistic regression was performed for all parameters. Subsequently, multivariate logistic regression was performed to find the optimal model for predicting post-operative atrial fibrillation. Akaike’s information criterion was used to optimize the models. The optimal multivariate model was based on parameters obtained using the ROC curve to achieve the highest possible value of area under the curve (AUC), sensitivity, and specificity.

## Results

### Patients and surgery

One hundred fifty patients were initially included in the study; however, 13 patients had to be excluded due to poor-quality ECG recordings. Ultimately, 137 patients (33 women and 104 men) were enrolled and analyzed. PoAF occurred in 48 patients (35%). These patients formed the AF group, and the remaining 89 formed the NoAF group. The baseline parameters of both groups are shown in Table 1. Regarding the clinical parameters, AF patients were significantly older (AF group 69.1 ± 8.6 vs. NoAF group 63.4 ± 10.5 years, p = 0.002) and had higher CHA_2_DS_2_-VASc scores (AF group 3 ± 1.4 vs. NoAF group 2.5 ± 1.3, p = 0.011), all other demographic, clinical and laboratory parameters were similar between groups.

**Table 1:** Baseline pre-operative characteristics of patients

Variable	AF Group (n = 48)	NoAF Group (n = 89)	Total (n = 137)	p
Gender, n (%)				
Female	15 (31%)	18 (20%)	33 (24%)	
Male	33 (69%)	71 (80%)	104 (76%)	0.15
Age, years mean ± SD	69.1 ± 8.6	63.4 ± 10.5	65.4 ± 10.2	**< 0.01**
BMI	30.2 ± 4.4	29.2 ± 5.2	29.55 ± 4.9	0.27
Ejection fraction (%) ± SD	55.7 ± 9.7	55.2 ± 8.9	55.4 ± 9.1	0.34
LVED (mm)	51 ± 6.5	51.4 ± 7.2	51.3 ± 6.9	0.75
Left atrial dimension (mm)	41.1 ± 5.6	39.4 ± 5.6	40 ± 5.6	0.09
Aortic valve disease, n (%)	15 (31%)	24 (27%)	39 (28%)	0.60
Mitral valve disease, n (%)	4 (8%)	7 (8%)	11 (8%)	0.67
Tricuspid valve disease, n (%)	1 (2%)	1 (1%)	2 (1%)	0.88
Diabetes mellitus, n (%)	17 (35%)	22 (25%)	39 (28%)	0.19
Heart failure, n (%)	2 (4%)	3 (3%)	5 (4%)	0.77
Thyroid disease, n (%)	7 (15%)	5 (6%)	12 (9%)	0.98
Coronary artery disease, n (%)	39 (81%)	74 (83%)	113 (82%)	0.78
Stroke/TIA, n (%)	3 (6%)	6 (7%)	9 (7%)	0.61
History of myocardial infarction, n (%)	13 (27%)	37 (42%)	49 (36%)	0.09
Hypertension, n (%)	38 (79%)	69 (78%)	107 (78%)	0.83
CHA2DS2-VASc scores	3 ± 1.4	2.5 ± 1.3	2.7 ± 1.4	**0.01**
**Medication on admission**				
Beta-blockers, n (%)	39 (81%)	69 (78%)	108 (79%)	0.61
Ca blockers, n (%)	8 (17%)	17 (19%)	25 (18%)	0.73
ACE inhibitors, n (%)	30 (63%)	58 (65%)	88 (64%)	0.76
**Blood count on admission**				
Leukocytes [x 10^5^/l]	8.7 ± 4.6	7.9 ± 1.9	8.2 ± 3.2	0.48
Na [mmol/l]	139.1 ± 2.8	138.6 ± 4.6	138.8 ± 4.1	0.60
K [mmol/l]	4.4 ± 0.4	4.3 ± 0.3	4.3 ± 0.3	0.36
Creatinine [µmol/l]	94.6 ± 25.3	91.1 ± 19.4	92.3 ± 21.5	0.69
Urea [mmol/l]	6.2 ± 2.8	5.5 ± 1.7	5.8 ± 2.2	0.10
C-reactive protein[mg/l]	6.3 ± 19.3	4.4 ± 5.6	5.1 ± 12.3	0.25
**Type of operation**				
CABG, n (%)	28 (58%)	60 (67%)	88 (64%)	0.29
Valve surgery, n (%)	11 (23%)	19 (21%)	30 (22%)	0.83
Combined (bypass + valve surgery), n (%)	9 (19%)	10 (11%)	19 (14%)	0.23

### Heart rate variability analysis

As mentioned, analyses of heart rate variability from 2-hour ECG recordings were performed. Time and frequency parameters, as well as non-linear parameters, were analyzed. The results are shown in Tables 2 and 3, and 4. In the time domain parameters analysis, the pNN50 (percentage of successive RR intervals that differ by more than 50 ms) was significantly lower in the AF group compared to the NoAF group.

**Table 2:** HRV – results of the time domain analysis

Parameter	AF Group (n = 48)	NoAF Group (n = 89)	p
Mean RR [ms]	920 ± 105	935 ± 127	0.47
Mean HR [beats/min]	66 ± 8	66 ± 9	0.63
RMSSD^*^ [ms]	24.1 ± 11.9	26 ± 9.6	0.18
pNN50^**^ [%]	3.8 ± 4.1	6 ± 5.5	**0.02**
TINN^***^ [ms]	171.7 ± 75.1	174.3 ± 88.4	0.87

^*RMSSD – Root mean square of successive differences between normal heartbeats^

^**pNN50 – Percentage of successive RR intervals that differ more than 50 ms^

^***TINN – Triangular interpolation of the NN Interval Histogram^

The results of the frequency domain analysis are shown in Table 3. In this analysis, two variables (1) absolute power in the VLF area (1524 ± 1118 ms^2^ in AF group vs. 1861 ± 1134 ms^2^in NoAF group, p = 0.04) and (2) total power (2118 ± 1675 ms^2^ in AF group vs. 2573 ± 1563 ms^2^ in NoAF group, p = 0.04), were significantly different between the AF and NoAF group.

**Table 3:** HRV - results of the frequency domain analysis

Parameter	AF Group (n = 48)	NoAF Group (n = 89)	p
Peak frequency VLF [Hz]	0.0053 ± 0.003	0.0055 ± 0.004	0.90
Peak frequency LF [Hz]	0.043 ± 0.001	0.043 ± 0.0003	0.15
Peak frequency HF [Hz]	0.228 ± 0.065	0.219 ± 0.06	0.48
Absolute power VLF [ms^2^]	1524 ± 1118	1861 ± 1134	**0.04**
Absolute power LF [ms^2^]	397 ± 438	494 ± 410	0.07
Absolute power HF [ms^2^]	195 ± 246	216 ± 175	0.07
Total power [ms^2^]	2118 ± 1675	2573 ± 1563	**0.04**
LF/HF^*^	2.73 ± 1.58	2.71 ± 1.35	0.72

^*The ratio of the low and high−frequency band power^

The results of the non-linear analyses are shown in Table 4. In the non-linear analyses, SD1 and SD2, and Guzik’s index were similar between both groups.

However, several other non-linear parameters differed significantly between the AF and non-AF groups (Table 4), i.e., Approximate Entropy (ApEn), Sample Entropy (SampEn), Correlation Dimension (D_2_), and recurrence rate (REC).

**Table 4:** Results of non-linear analysis

Parameter	AF Group (n = 48)	NoAF Group (n = 89)	p
SD1 [ms]	17.04 ± 8.4	18.4 ± 6.8	0.18
SD2 [ms]	59.36 ± 22.68	67.21 ± 21.13	**0.05**
SD1/SD2	0.288 ± 0.093	0.277 ± 0.079	0.50
ApEn	1.11 ± 0.17	1.17 ± 0.15	**0.03**
SampEn	1.1 ± 0.23	1.19 ± 0.23	**0.04**
D_2_	1.028 ± 0.58	1.52 ± 0.9	**0.01**
REC [%]	46.3 ± 5.2	44.2 ± 4.6	**0.02**
Guzik index (%)	49.9 ± 0.04	50 ± 0.04	**0.05**
Porta index (%)	50.6 ± 2.3	50.1 ± 1.87	0.17
Slope index (%)	52.87 ± 1.26	52.43 ± 0.99	0.06

### Logistic regression

**Fig. 1:**
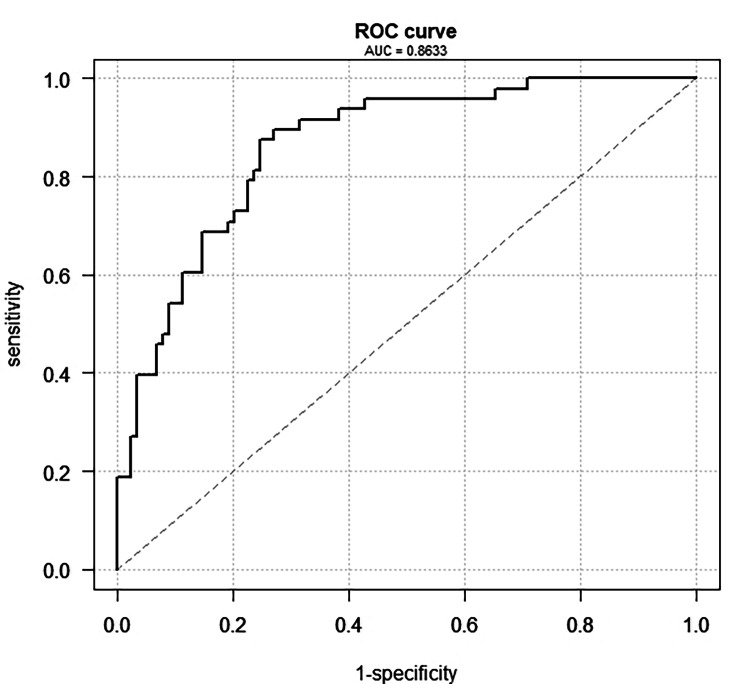
ROC curve – optimal multivariant logistic model

First, a univariate logistic regression was performed for all parameters. The following parameters were independent predictors of post-operative atrial fibrillation: age, CHA2DS2-VASc, CHA2DS2-Risc, NN50, pNN50, SD2, ApEn, Slope index, SampEn, D2, and REC. Odds ratios and p values are shown in Table 5. Additionally, several multivariate logistic models were assessed. Only variables significantly associated with AF in the univariate analysis were included in the first model. This model had an AUC of 0.71, a sensitivity of 0.95, and a specificity of 0.29, and of the parameters included, only D2 reached the significance threshold. After optimizing the model using Akaike’s information criterion, a slightly higher specificity was achieved (0.3), and statistical significance was reached by D2 and age (p = 0.01 and 0.02, respectively). Due to the low specificity value, another logistic model was calculated using only parameters from the HRV analysis (except for those that were strongly correlated) but without clinical variables. After optimization, this second model consisted of pNN50, SD2, SD1/SD2, the Porta index, the Guzik index, and the Slope index. All mentioned parameters except for the Porta index reached statistical significance. The parameters of the ROC curve were as follows: AUC 0.75, sensitivity 0.95, and specificity 0.29; i.e., specificity was also very low in the second model. Therefore, the third model was done that consisting of parameters from the HRV analysis and clinical variables selected in the first model, was tested. Using Akaike’s optimization, this model achieved the highest ROC curve values: AUC 0.86, sensitivity 0.95, and specificity 0.57 (see Fig. 1). Therefore, this model was chosen as the optimal one. The parameters included in the model and their odds ratios with p values are shown in Table 6.

Correlations between individual parameters that were used in multivariate logistic regression were tested. The results of the most significant correlations are shown in Table 7. The most significant correlation was between Absolute power in the VLV area and Total power. Total power is the sum of the powers in the VLF, LF, and HF areas.

**Table 5:** Results of univariate logistic regression

Parameter	Odds ratio	p
Age	1.07	0.01
CHA2DS2-VASc	1.32	0.04
NN50	0.99	0.03
pNN50	0.91	0.02
SD2	0.98	0.05
ApEn	0.09	0.03
Slope index	1.43	0.03
SampEn	0.19	0.04
D2	0.43	0.001
REC	1.1	0.02

**Table 6:** Results of the optimal multivariate logistic model

Parameter	Odds ratio	p
LVED	0.91	0.09
LAD	1.15	0.03
Hypertension	0.27	0.06
Diabetes mellitus	4.69	0.009
Thyroid disease	6.22	0.02
History of myocardial infarction	0.17	0.003
COPD	5.24	0.03
Beta-blockers	4.45	0.03
Leukocytes	1.26	0.004
Na	1.19	0.10
C-reactive protein	1.04	0.04
Mean RR	1.01	0.04
pNN50	0.70	0.03
TINN	1.01	0.08
Absolute power VLF	0.25	0.03
Absolute power LF	0.25	0.03
Absolute power HF	0.25	0.03
Total Power	3.96	0.03
LF/HF	1.71	0.04
SD2	0.86	0.03
Porta Index	1.28	0.07

**Table 7:** The largest correlation coefficients between the parameters used in the multivariate logistic model

Parameter 1	Parameter 2	Correlation coefficients
Absolute power VLF	Total power	0.96
Absolute power VLF	SD2	0.88
Absolute power LF	Total power	0.84
Absolute power LF	SD2	0.77
Absolute power HF	Total power	0.74
pNN50	Total power	0.64

## Discussion

Post-operative atrial fibrillation after cardiac surgery occurred in 35% of patients in our study. Regarding clinical parameters, older age and higher CHA2DS2-VASc scores were associated with the presence of PoAF. In the univariate logistic analysis of HRV parameters, NN50, pNN50, SD2, ApEn, Slope index, SampEn, D2, and REC were independent predictors of PoAF. In the multivariate logistic regression, a combination of several clinical and HRV parameters was able to predict PoAF. According to the ROC analysis, the highest specificity was achieved using pNN50, Absolute power in VLF, LF, and HF area, Total power, LF/HF, and SD2 from the HRV variables, and diabetes, history of thyroid disease, and COPD from the clinical variables.

PoAF in 35% of patients is consistent with results from other studies. Depending on the type of procedure, post-operative fibrillation occurs in 20 to 60% of patients. Rader [13] also reported PoAF in 35% of patients. Also, this study included patients who underwent isolated coronary artery bypass grafting, isolated valve surgery, or both. Pollock [14] focused only on patients undergoing CABG. They reported PoAF in 24% of patients. This was in agreement with our results, i.e., in our CABG patients, AF appeared in 32% (28) of patients.

The exact cause of post-operative atrial fibrillation is not known. The etiology is definitively multifactorial, with several risk factors. One of the most important risk factors is older age at surgery [4, 15]. In accordance with results from previous studies, our study patients who experienced PoAF were significantly older (p = 0.002) than those without PoAF.

Another critical factor is the CHA_2_DS_2_-VASc score, which comprehensively describes the patient´s risk profile. Chua [16] found that the risk of PoAF increases with increasing CHA2DS2-VASc scores. Similarly, in our study, patients with PoAF had higher CHA2DS2-VASc scores than NoAF patients (AF group 3 ± 1.4 vs. NoAF group 2.5 ± 1.3, p = 0.011). The univariate regression also shows that the risk of PoAF increases with increasing CHA2DS2-VASc score.

Regarding other clinical variables in the multivariate model, a history of thyroid disease, diabetes, and COPD independently predicted the risk of PoAF. The relationship between thyroid disease and the occurrence of atrial fibrillation is well established; moreover, patients with no signs of acute hyperthyroidism but with a history of thyroid disease are also at a higher risk of AF [17].

Various studies found that patients with diabetes have a 34% greater risk of developing AF than control subjects without diabetes. Diabetes is accompanied by structural, electrical, electromechanical, and autonomic activation of the atria; all increase the risk of AF [18]. Not surprisingly, COPD was also associated with a higher risk for PoAF in our study since AF is the most common arrhythmia in COPD patients [19].

The main goal of this study was to assess whether parameters obtained from a heart rate variability analysis could predict the occurrence of PoAF. It has to be emphasized that it is difficult to compare the results of heart rate variability analysis between different studies: to compare the results of individual studies, the ECGs would have to be recorded under precisely the same conditions for the same amount of time.

Parameters from the time domain are the most intuitive and the easiest to calculate. The most representative of this cluster is heart rate which did not differ between the groups. The time-domain parameter that differed significantly (and also reached significance in the multivariate model) between the groups was pNN50 (3.8 ± 4.1% AF group vs. 6 ± 5.5% NoAF group, p = 0.02). pNN50 is closely correlated with parasympathetic nervous system activity, and higher RR oscillations, a measure of higher vagal tone, are associated with a lower risk of PoAF. In a report by Chamchad et al. [20], the average pNN50 value was also slightly (optically) lower in patients with PoAF, but it did not reach statistical significance. An explanation might be related to the length of the ECG recording. Chamchad et al. did only 10-min ECG recordings, but more importantly, the recordings were obtained just before anesthesia. Our recordings were substantially longer and were done one day before surgery and before any pre-operative medications were given. Therefore, and not surprisingly, our results are similar to those reported by Kališnik et al. [21], who also recorded ECGs one day before surgery. In their report, higher levels of pNN50 were also associated with a lower risk of PoAF.

Regarding the parameters obtained from the frequency analysis, low values of the absolute power in the VLF area were significantly associated with a higher risk of PoAF. Furthermore, total power was significantly lower in patients with PoAF (2118 ± 1675 ms^2^ in the AF group vs. 2573 ± 1563 ms^2^ in the NoAF group). Previous studies have shown that low VLF power is associated with arrhythmic death. In other studies, inflammation was associated with a decrease in the VLF area [8]. The absolute levels in the VLF, LF, and HF region, the power (assumed to be total power), was lower in patients with PoAF. Thus, decreased total power in these regions seems to make the patients more susceptible to PoAF, i.e., patients with lower heart rate variability are at a higher risk of PoAF. According to our logistic model, the risk of post-operative atrial fibrillation increases as the LF/HF ratio increases. Several lines of evidence and studies support the HF component’s vagal origin. In contrast, the interpretation of the LF component is somewhat more complicated. It is assumed that LF power can be produced by parasympathetic and sympathetic systems and blood pressure regulated through baroreceptors. Therefore, it is impossible to interpret this parameter as an indicator of sympathovagal balance, as before [22].

Non-linear methods of HRV analysis have been shown, in some studies, to be a helpful tool providing important information about the risk of ventricular arrhythmias [23, 24] and are increasingly used in atrial fibrillation analysis [25]. Results from the non-linear region revealed that most parameters differed significantly between groups in our study. These parameters were also crucial in the univariate and multivariate analysis.

The classic Poincare plot is often used to measure the non-linear regulation of cardiovascular control. Two measures of standard deviation are most often used to describe the graph. SD1 is a measure of variability across the line of identity that indicates how big the difference in duration is between two consecutive intervals. The SD2 parameter is used as a measure of variability along the line of identity, indicating how interspersed consecutive RR intervals of equal or similar duration are [26]. It has been shown that the SD1 and SD2 indexes represent more or less linear features of heart rate dynamics [27]. The second parameter, SD2, was lower in patients with PoAF, and SD2 was also an independent predictor in the univariate and multivariate logistic regression. In previous studies, the SD2 parameter correlates with LF and HF power and the baroreflex sensitivity [8, 27].

Another parameter showing the complexity or strangeness of the time series was correlated with the D_2_ dimension. The correlated dimension gives information regarding the minimum number of dynamic variables needed to model the underlying system [7]. The more variables required to predict the time series, the greater the complexity [8]. Since the D2 value was lower in the patients who developed PoAF, it again shows the attenuation of HRV in patients at high risk of PoAF, much as it did in the analysis of linear parameters.

Recurrence is a fundamental property of dynamic systems, which can be exploited to characterize the system’s behavior in phase space. A powerful tool for visualization and analysis is a recurrence plot [28]. Our data show that as the REC (OR 1.1 in the univariate analysis) increases, the chance of developing post-operative atrial fibrillation increases.

Approximate entropy measures the regularity and complexity of a time series. Applied to HRV data, large ApEn values indicate low predictability of fluctuations in successive RR intervals. Small ApEn values mean that the signal is regular and predictable [8]. Several studies analyzed heart rate complexity before the initiation of paroxysmal atrial fibrillation. ApEn and SampEn were reported to decrease significantly before atrial fibrillation onset. These results indicate that decreasing ApEn prior to the onset of atrial fibrillation indicates that the heart rate becomes more ordered before an AF paroxysm. In other words, there is some loss of healthy complexity that enables AF to initiate [25–29]. Hogue et al. [30] showed that lower ApEn was independently associated with PoAF in patients after CABG, which agrees with our results. In our study, ApEn was significantly lower in patients who experienced PoAF, and low ApEn and SampEn remained independent predictors of PoAF in the multivariate analysis. This again emphasizes that attenuation of variability increases the risk of AF. SampEn is measured in the same way as ApEn but is less dependent on the length of the time series [31]. SampEn results are interpreted the same way as ApEn results and confirm that patients with complexity attenuation are at higher risk of PoAF.

The Guzik and Slope index are obtained from Poincare plots and are used to describe the level of heart rate asymmetry [11]. These parameters also prove to be helpful in detecting patients at risk of atrial fibrillation. A Guzik index value greater than 50 (resp. less than 50) means the presence of asymmetry in HRV dynamics. In this case, accelerating (resp. decelerating) dynamics prevail over decelerating (resp. accelerating) HRV dynamics [32]. In our cohort, Guzik’s index was slightly lower in PoAF patients, but this difference had only borderline significance (p = 0.05). The Slope index emerged as an independent predictor in the univariate analysis; however, similar to Guzik´s index, it was only borderline significant in the multivariate analysis. (p = 0.06).

### Study limitations

In the study, 137 patients were enrolled. The number of patients in our study was limited and therefore did not allow clear conclusions of the effect of different parameters of HRV on the prediction of postoperative AF. Parameters found to be significant predictors should be further tested in more extensive studies. Longer ECG recordings (optimally 24 h) would be useful and allow more detailed HRV analysis.

Patients on different types of beta-blockers were included in the study. An analysis of patients on and off beta-blockers found no differences; however, substantially more patients would be needed for a complete comparison.

Our study focused on patient demographics, medical history, and especially HRV parameters that could predict postoperative AF. However, other peri- and early postoperative parameters, such as the use of cardiopulmonary bypass, the length of cross-clamp, surgery duration, and need for inotropes after cardiac surgery also play a role in the risk of postoperative fibrillation; however, these additional parameters were not analyzed in our study.

## Conclusion

The cardiovascular system is a complex, non-linear and non-stationary system, the analysis of which constantly brings new information. This heart rate variability study shows that non-linear methods hold good promise for analyzing the underlying principles.

Various HRV parameters of time, frequency, and non-linear analysis were found to be statistically different between the investigated groups. Furthermore, two models were created using logistic regression. If we include demographic data in the HRV analysis parameters, we get a more accurate model with an AUC of 0.86. However, the model-based only on HRV analysis parameters also achieved good sensitivity and specificity values. Our results, after validation, could help successfully identify patients with a higher risk of post-operative atrial fibrillation and better prophylaxis.

## Data Availability

The datasets used and analysed during the current study available from the corresponding author on reasonable request.

## References

[1] ZAKKAR M, ASCIONE R, JAMES AF, ANGELINI GD. and M.S. SULEIMAN. Inflammation, oxidative stress and post-operative atrial fibrillation in cardiac surgery. *Pharmacology& Therapeutics* 2015, 154(October 2015), 13–20. DOI: 10.1016/j.pharmthera.2015.06.009. ISSN 01637258. 10.1016/j.pharmthera.2015.06.009 26116810

[2] MAESEN B, NIJS J, J. MAESSEN M, ALLESSIE (2012). Post-operative atrial fibrillation: a maze of mechanisms. Europace.

[3] Ji SEOE, Joonhwa HONG, Hyeon-Ju LEEa, Youn-Jung SON (2021). Perioperative risk factors for new-onset post-operative atrial fibrillation after coronary artery bypass grafting: a systematic review. BMC Cardiovasc Disord.

[4] Hristo TODOROV, Inka JANSSEN, Stefanie HONNDORF (2017). Clinical significance and risk factors for new onset and recurring atrial fibrillation following cardiac surgery - a retrospective data analysis. BMC Anesthesiol.

[5] Hugh CALKINS, Gerhard HINDRICKS, Riccardo CAPPATO (2018). 2017 HRS/EHRA/ECAS/APHRS/SOLAECE expert consensus statement on catheter and surgical ablation of atrial fibrillation: executive summary. EP Europace.

[6] ELECTROPHYSIOLOGY TF, Clinical Use. o. t. E. S. o. C. t. N. A. S. Heart Rate Variability: Standards of Measurement, Physiological Interpretation, and. *Circulation* [online]. 1996, 93(5), 1043–1065. ISSN 0009-7322. 10.1161/01.CIR.93.5.1043. 8598068

[7] HRV analysis methods. *Kubios* [online]. Kuopio: Kubios Oy, c2022. Available from: https://www.kubios.com/hrv-analysis-methods/.

[8] Fred a SHAFFER (2017). GINSBERG. An overview of Heart Rate Variability Metrics and norms. Front Public Health.

[9] RAJENDRA ACHARYA, U. K, PAUL JOSEPH NKANNATHAL, Choo Min LIM, a Jasjit. S. SURI. *Heart rate variability: a review*. 2006, 44(12), 1031–1051. ISSN 0140 – 0118. 10.1007/s11517-006-0119-0. 10.1007/s11517-006-0119-0 17111118

[10] Akemi HOSHIR, Carlos Marcelo PASTRE, Luiz Carlos Marques VANDERLEI a, Moacir Fernandes GODOY. Poincaré plot indexes of heart rate variability: Relationships with other non-linear variables. *Autonomic Neuroscience*. 2013, 177(2), 271–274. ISSN 15660702. 10.1016/j.autneu.2013.05.004. 10.1016/j.autneu.2013.05.004 23755947

[11] KARMAKAR CK, AH, KHANDOKER a M, PALANISWAMI (2015). Phase asymmetry of heart rate variability signal. Physiol Meas.

[12] PORTA A, CASALI KR, CASALI AG (2008). Temporal asymmetries of short-term heart period variability are linked to autonomic regulation. Am J Physiology-Regulatory Integr Comp Physiol.

[13] Florian RADER, Otto COSTANTINI, Craig JARRETT, Eiran Z, GORODESKI, Michael S, LAUER a Eugene H (2011). BLACKSTONE. Quantitative electrocardiography for predicting post-operative atrial fibrillation after cardiac surgery. J Electrocardiol.

[14] Giovanni POLLOCKBD, Briget FILARDO, Teresa DAGRACA, PHAN K, Gorav AILAWADI, Vinod THOURANI, Ralph J, DAMIANO (2018). JR a James R. EDGERTON. Predicting New-Onset Post-Coronary Artery Bypass Graft Atrial Fibrillation with existing risk scores. Ann Thorac Surg.

[15] OVREIU MIRELA, NAIR BALAG, MENG XU (2008). Electrocardiographic Activity before Onset of Postoperative Atrial Fibrillation in Cardiac surgery patients. Pacing and Clinical Electrophysiology [online].

[16] Su-Kiat CHUA, Kou-Gi SHYU, Ming-Jen LU, Li-Ming LIEN, Chia-Hsun LIN, Hung-Hsing CHAOa, Huey-Ming LO (2013). Clinical utility of CHADS2 and CHA2DS2-VASc scoring systems for predicting post-operative atrial fibrillation after cardiac surgery. J Thorac Cardiovasc Surg.

[17] Alexandra BEKIARIDOU, Anastasios KARTAS, Dimitrios VMOYSIDIS, Andreas S, PAPAZOGLOU, Amalia BAROUTIDOU, Anastasios PAPANASTASIOU, a George GIANNAKOULAS (2022). The bidirectional relationship of thyroid disease and atrial fibrillation: established knowledge and future considerations. *Reviews in endocrine & metabolic disorders* [online].

[18] WANG A, GREEN JB, Jonathan L, HALPERIN a Jonathan P. PICCINI. Atrial Fibrillation and Diabetes Mellitus: JACC Review Topic of the Week. *Journal of the American College of Cardiology* [online]. NEW YORK: Elsevier, 2019, 74(8), 1107–1115. ISSN 0735–1097. 10.1016/j.jacc.2019.07.020. 10.1016/j.jacc.2019.07.020 31439220

[19] HUANG, Qiangru, Huaiyu XIONG, Tiankui SHUAI et al. Risk factors for new-onset atrial fibrillation in patients with chronic obstructive pulmonary disease: A systematic review and meta-analysis. *PeerJ (San Francisco, CA)* [online]. United States: PeerJ., 2020, 8, e10376-e10376. ISSN 2167–8359. 10.7717/peerj.10376 PMC7718784 33344074

[20] Dmitri CHAMCHAD, HORROW JC, Louis E, SAMUELS a Lev NAKHAMCHIK (2011). Heart rate variability measures poorly predict atrial fibrillation after off-pump coronary artery bypass grafting. J Clin Anesth.

[21] KALIŠNIK, Jurij M, Viktor AVBELJ, Jon VRATANAR, Giuseppe SANTARPINO, Borut GERÅAK, Theodor FISCHLEIN, Roman TROBEC, a Janez ŽIBERT (2019). Cardiac autonomic regulation and PR interval determination for enhanced atrial fibrillation risk prediction after cardiac surgery. Int J Cardiol.

[22] REYES DEL PASO, Gustavo A, Wolf LANGEWITZ, Lambertus JM, MULDER, Arie VAN, ROON a Stefan DUSCHEK (2013). The utility of low frequency heart rate variability as an index of sympathetic cardiac tone: a review with emphasis on a reanalysis of previous studies. Psychophysiol [online] HOBOKEN: Blackwell Publishing.

[23] SKINNER JE, Craig M, PRATT a Tomas VYBIRAL (1993). A reduction in the correlation dimension of heartbeat intervals precedes imminent ventricular fibrillation in human subjects. Am Heart J.

[24] FLEISHER LA, PINCUS a SM (1993). ROSENBAUM. Approximate entropy of heart rate as a correlate of post-operative ventricular dysfunction. *Anesthesiology (Philadelphia)* [online].

[25] Ernie LIAOTing-Wei, Li-wei LO, Yenn-jiang LIN (2022). Nonlinear Heart Rate Dynamics before and after paroxysmal atrial fibrillation events. *Acta Cardiologica Sinica* [online]. Taiwan Soc Cardiol.

[26] PLATIŠA MM, Tijana BOJIC, Siniša U, PAVLOVIC, Nikola N, RADOVANOVIC a Aleksandar KALAUZI (2016). Generalized Poincaré plots-A new method for evaluation of regimes in cardiac neural control in atrial fibrillation and healthy subjects. *Frontiers in neuroscience* [online]. LAUSANNE: Front Media.

[27] BARBIERI R, Pasquale E. SCILINGO a, Gaetano VALENZA. *Complexity and Nonlinearity in Cardiovascular Signals*. Cham: Springer International Publishing, 2017, 1 online resource (537 pages). ISBN 3-319-58709-9. 10.1007/978-3-319-58709-7.

[28] MARWAN, Norbert MCARMENROMANO, Marco THIEL, a Jürgen. KURTHS. Recurrence plots for the analysis of complex systems. *Physics reports* [online]. AMSTERDAM: Elsevier B.V, 2007, 438(5), 237–329. ISSN 0370–1573. 10.1016/j.physrep.2006.11.001.

[29] Dong-gu SHIN, Sang-hoon Cheol-seungYOO, Jun-ho YI, Young-jo BAE. KIM, Jong-sun PARK a Geu-ru HONG. Prediction of Paroxysmal Atrial Fibrillation Using Nonlinear Analysis of the R-R Interval Dynamics Before the Spontaneous Onset of Atrial Fibrillation. *Circulation journal: official journal of the Japanese Circulation Society* [online]. Japan: The Japanese Circulation Society, 2006, 70(1), 94–99. ISSN 1346–9843. 10.1253/circj.70.94. 10.1253/circj.70.94 16377931

[30] Peter HOGUECW, Phyllis PDOMITROVICH, STEIN K, George D, DESPOTIS, Lisa RE, Richard B, SCHUESSLER, Robert E (1998). KLEIGER a Jeffery N. ROTTMAN. RR interval Dynamics before Atrial Fibrillation in Patients after coronary artery bypass graft surgery. Circulation.

[31] Heikki HUIKURI, Juha V, Roberto SPERKIÖMÄKI, a Gian Domenico MAESTRI (2009). Clinical impact of evaluation of cardiovascular control by novel methods of heart rate dynamics. *Philosophical transactions of the Royal Society of London. Series A: Mathematical, physical, and engineering sciences* [online].

[32] ALVAREZ-RAMIREZ J, ECHEVERRIA JC, MERAZ a E M. RODRIGUEZ. Asymmetric acceleration/deceleration dynamics in heart rate variability. *Physica A* [online]. AMSTERDAM: Elsevier B.V, 2017, 479, 213–224. ISSN 0378–4371. 10.1016/j.physa.2017.03.008.

